# Temporal dynamics of neural activity in autism: dynamical systems perspective on sensitivity, neural learning, and social interactions

**DOI:** 10.3389/fnins.2025.1711892

**Published:** 2026-01-20

**Authors:** Evie A. Malaia, Sungwoo Ahn, Leonid L. Rubchinsky

**Affiliations:** 1 Department of Speech, Language and Hearing, University of Alabama, Tuscaloosa, AL, United States; 2 Department of Mathematics, East Carolina University, Greenville, NC, United States; 3 Alliance for Brain Stimulation, College of Allied Health Sciences, East Carolina University, Greenville, NC, United States; 4 Department of Mathematical Sciences, Indiana University, Indianapolis, IN, United States; 5 Stark Neurosciences Research Institute, Indiana University School of Medicine, Indianapolis, IN, United States

**Keywords:** ASD, autism, EEG, neural oscillations, neural synchronization, nonlinear dynamics, resting state, state transitions

## 1 Introduction

A dynamical systems approach to the analyses of neural data shows that autism spectrum disorder (ASD) is characterized by specific temporal patterning of the neural activity that may affect the sensitivity of brain networks to input signals. Changes in the responsiveness of a network on a short-term timescale may lead to changes in the network's behavior on longer timelines and in input-based learning mechanisms such as habituation and predictive processing. In this opinion paper, we discuss recent experimental results on the temporal patterning of the neural activity in autism, consider their potential consequences, and review what experimental and theoretical developments may help advance the neural mechanistic understanding of autism spectrum disorder.

## 2 Heterogeneity in ASD

The single, deficit-based model of autism has recently come under scrutiny, as research revealed subgroups differing in symptoms, developmental trajectory, and genetic drivers of the disorder (Litman et al., 2025). Instead, questions can be directed to specific ASD subtypes to examine qualitatively distinct neural processing mechanisms and characteristics of each subtype. The classical neuroscience framework, the Hebbian principle that neurons, which ‘fire together, wire together'—i.e., that synaptic connections get modified when pre- and post-synaptic neurons are active at the appropriate time with respect to each other—might, in fact, manifest differently depending on the temporal (as well as spatial) patterning of network organization.

Low-support needs subgroup of ASD shows intact within-group communication (Crompton et al., 2025; Wilks et al., 2025), suggesting that communication challenges emerge specifically due to mismatches in neurotype. But what do neurotype mismatches consist of, and how do they emerge over time? Recent research suggests that autistic brains adopt different adaptive strategies for processing environmental inputs and social information, characterized by increased network-engagement variability among multiple trials and enhanced sensitivity to inputs (Barnes et al., 2025; Malaia et al., 2020). A more fundamental question is, however—how do these differences emerge and what neurophysiological dynamic mechanism may underly them?

## 3 Characterizing temporal dynamics of neural activity

Conventional time- and frequency-based measures of neurodynamics and functional connectivity in ASD, such as the characteristics of the power spectrum or time-average correlations and spectral-based coherence which rely on time-averaging and have poor temporal resolution, tend to show minimal (Li et al., 2022) and/or inconsistent (Liang and Mody, 2022; Soto-Icaza et al., 2024) differences between diagnosed and neurotypical individuals. These analyses quantify neural activity by averaging features under investigation across extended time windows (several seconds to minutes), and thus obscuring the rapid, moment-to-moment fluctuations in neural coordination. On the other hand, the study of temporal patterns of neural activity reveals fine-grained temporal differences that are invisible to conventional measures. For example, Malaia et al. (2020) demonstrated that adolescents with ASD exhibit higher frontoparietal synchronization accompanied by more frequent short periods of desynchronization, as measured by increased desynchronization ratio (DR, a measure that characterizes the relative prevalence of the brief transitions between desynchronized and synchronized states, Ahn et al., 2014). This pattern indicates more frequent transitions between synchronized and desynchronized states across brain regions, which, according to mathematical modeling (Ahn and Rubchinsky, 2017; Nguyen and Rubchinsky, 2021, 2024), may be facilitated by cellular and synaptic changes and may facilitate network's dynamics with altered (likely elevated) sensitivity to inputs. Note that the changes in the excitatory/inhibitory balance, that is known to modulate synchrony in cortical circuits in general (Wagatsuma et al., 2025) and is implied in ASD (Gao and Penzes, 2015; Sohal and Rubenstein, 2019), may, according to the numerical simulations in Nguyen and Rubchinsky (2021, 2024) also affect the temporal patterning of synchronous activity.

Ahn et al. (2025b) explored distinct patterns of brain activity in autistic young adults compared to neurotypical peers, using dynamical systems analysis of resting-state EEG. These methods include measures that characterize the instabilities of dynamics (such as Lyapunov exponents) and measures focused on the properties of the temporal patterning of the dynamics (characteristics of transitions between synchronized and desynchronized states) and in that way they are different from the power spectrum or spectral coherence measures that inherently dismiss the details of the temporal variability. While some EEG measures based on time-averaged characteristics (and thus with low temporal resolution) showed minimal differences between ASD and neurotypical groups, other measures, dynamic measures and measures that are focused on the higher temporal resolution (such as the measures of the temporal patterning of synchrony, Ahn et al., 2011, 2018; Ahn and Rubchinsky, 2013), indicated that autistic participants exhibited less stable neural dynamics and weaker functional network connectivity when their eyes were open, with the effect primarily localized to frontal cortical region. Notably, in eyes-closed resting state, the same participants were able to stabilize their neural activity, leading to increased inter-regional synchrony and brain network connectivity (no change of this type was observed in neurotypical participants). These findings indicate a mechanistic trade-off between sensitivity and stability of brain network activity, with a tendency toward sensitivity (over stability) in ASD. It should be noted that while closing the eyes typically results in substantial increases in occipital alpha activity, the effects reported here were primarily localized to anterior (frontal) cortical regions and are therefore distinct from the well-established posterior alpha potentiation related to eye-closed condition. The less stable neural systems may habituate more poorly because frequent transitions between different states may prevent stable learning: the system remains “fresh” to repeated inputs, preventing adaptive signal suppression. In a general way, these observations and considerations are supported by earlier studies of the properties of the microstates, spatiotemporal patterns that are altered in ASD (Das et al., 2002).

One can (and perhaps should) consider taking a cautious outlook here as the experimentally observed differences between ASD and neurotypical individuals in the measures of the temporal dynamics of the neural activity are relatively modest. Short desynchronizations *per se* are common in healthy brain and different disease states (Ahn and Rubchinsky, 2013; Rubchinsky et al., 2012; Ahn et al., 2025a). However, this kind of situation has been argued to be quite expected and observed in different experimental situations (Ahn et al., 2018). It may be possible to use some data measures to artificially generate large differences between different conditions. Yet it is important to focus on the measures that are characteristic of the neurophysiological mechanisms and small differences in numerical measures may just point to the relatively similar functionality of healthy and diseased states.

## 4 Extending the timescale: learned responses to sensory inputs

How might highly sensitive but unstable brain networks learn over time, and how might this affect behavior in ASD individuals? Frequent state transitions would allow for rapid reconfiguration of neural networks in response to external stimuli. This increased sensitivity, over time, might lead to different results via input-based learning mechanisms: a neurophysiological organization that may explain both the challenges and strengths observed in ASD participants.

One such challenge in autistic individuals is relative lack of habituation, or diminished decrease in response to repeated stimuli. Several fMRI studies showed that individuals with autism spectrum disorder show diminished habituation to repeated social and non-social stimuli (Kleinhans et al., 2009; Swartz et al., 2013); notably, attenuation levels of habituation correlated with ASD severity, as measured by standard ASD diagnostic tools—Social Responsiveness Scale and ADOS scores. EEG research, which provided higher temporal resolution, repeatedly indicated that ASD participants have a delayed attenuation for ERP components elicited by repeated stimuli in both auditory and visual modalities (Dwyer et al., 2023; Jamal et al., 2020). The same studies showed that the delay in habituation effects was significantly correlated with both sensory sensitivity and measures of social communication.

While the relationship between temporal patterning of neural activity and longer-term learning mechanisms such as habituation is, at the moment, speculative, computational modeling suggests potential mechanistic links between the two. Spike-timing dependent plasticity, a fundamental form of synaptic plasticity, has been shown to promote short desynchronization dynamics in neural networks (Zirkle and Rubchinsky, 2020). Such plasticity-driven temporal patterning could potentially contribute to the frequent state transitions that may underlie poor habituation in ASD. The theoretical mechanistic link between cellular-level mechanisms and behavioral outcomes requires, however, further experimental investigation.

## 5 Implications for social and cognitive functioning in ASD

The temporal volatility characteristic of autistic neural dynamics may have profound implications for learning mechanisms, which are fundamentally predictive. Temporal instability in network organization could result in learning of predictive models heavily weighted toward recent information, potentially explaining the documented difficulties with habituation and adaptation to environmental regularities in autistic participants.

The temporal volatility and enhanced input sensitivity characteristic of autistic neural dynamics may also directly impact real-time social interactions between autistic and neurotypical individuals, as recent hyperscanning studies of live social interactions show. For example, Key et al. (2022) showed that although adolescents with ASD had increased interpersonal neural synchrony during conversation compared to baseline, the synchrony was significantly associated with autism symptom severity. Lower levels of synchrony correlated with more behavioral symptoms of social difficulties, suggesting that the frequent state transitions and temporal instability identified in resting-state studies may translate into measurable deficits during social engagement. Similarly, Wang et al. (2020) found that children with ASD showed increased interpersonal neural synchronization in frontal cortex during cooperative interactions with parents, but this synchronization, again, was modulated by autism symptom severity: children with more severe autism symptoms had lower levels of both action synchronization and neural synchronization with their parents during cooperation tasks. These findings are complemented by a recent fMRI hyperscanning study that used state-space modeling of dynamic functional connectivity during live social exchanges (Czekóová et al., 2025). This study found that autistic adults exhibited reduced expressions of reciprocity during an interactive economic game, and that this behavioral pattern was associated with altered temporal dynamics of latent brain states. It is notable that autistic participants spent less time in states characterized by dynamic integration and segregation among the default mode network and cognitive control networks. Converging evidence from neuroimaging modalities strengthens the view that temporal neural dynamics form the mechanistic basis for social interaction differences in autism.

These quantitative observations in live interactions provide a connection between the temporal patterning differences observed at the individual level in resting state data, and the social communication challenges in interpersonal interactions. The frequent desynchronization events and enhanced sensitivity that characterize autistic neural dynamics at rest may create a fundamental mismatch in the temporal coordination required for successful social synchrony. This provides for a hypothetical mechanistic framework connecting the very quick and dynamics changes and learned responses to the sensory inputs (as discussed in the previous section) to the behavioral aspects of social interaction.

The symptom severity-based convergence between individual neural temporal pattern structures and dyadic synchronization measures suggests that neural coordination provides the basis for successful social interaction. This evidence supports the hypothesis that neurotype mismatches in communication arise not just from different processing styles, but from fundamental differences in the temporal organization of neural activity that develop into interpersonal coordination difficulties. Current findings on successful autistic peer-to-peer communication (Crompton et al., 2020, 2025; Wilks et al., 2025) can be better understood through the lens of neural temporal compatibility—matching of the quick dynamics changes in the networks facilitating the reactions of the networks to the input [and the aforementioned modeling results (Nguyen and Rubchinsky, 2024) may be viewed as a generally supporting argument]. When two individuals share similar patterns of temporal neural dynamics—for example, both participants in a conversation can be characterized, neurally, by high desynchronization ratios and enhanced input sensitivity—one might have an easier time replicating the mental model of the other. This is further supported fMRI data from Czekóová et al. (2025), as well as by Wan et al. (2024), that, in turn, appear to match the electrophysiological observations in Ahn et al. (2025b) in regard to the transitions between brain network states. Essentially, neurological similarity could facilitate more effective information transfer and mutual comprehension.

Conversely, communication breakdowns between autistic and neurotypical individuals (Crompton et al., 2020) may arise from mismatches in temporal and network-level processing patterns. Neurotypical individuals, with different temporal integration windows, may have difficulty adapting to the rapid state transitions and enhanced sensitivity characteristic of autistic neural dynamics.

## 6 Discussion

Critical gaps remain in our understanding of how temporal neural dynamics change across development in autism. One currently considered possibility is that repeated neurotype-mismatched interactions (e.g., between an autistic person and multiple neurotypical peers) may lead to the development of compensatory strategies that have long-term consequences.

The empirical evidence reviewed here includes child, adolescent, and adult samples, although with different types of data and analyses approaches. Thus, rather than treating neural differences as static features, we propose a mechanistic account of how brain networks in the low-support-needs ASD subgroup would self-organize over time: the elevated sensitivity and frequent state transitions we observe may reflect an adaptive system that, through processes like spike-timing dependent plasticity (Zirkle and Rubchinsky, 2020), develops distinct temporal coordination patterns that cascade into characteristic behavioral profiles. This perspective aligns with recent calls to place neurodevelopmental differences within their dynamic context, understanding divergence as an emergent phenomenon, rather than a fixed one (Astle et al., 2024). Microstate analyses of EEG support this developmental framing, with meta-analytic evidence (Wei et al., 2025) indicating age-dependent heterogeneity in temporal parameters, and work in young children (Bochet et al., 2021) demonstrating that alterations in large-scale network temporal dynamics are present early in development.

Comprehensive research measuring brain activity throughout lifespan using modalities with high spatiotemporal resolution is needed to bridge the gap between short and long timescales in neural development in ASD, and to develop a practically useful mechanistic framework applicable to therapeutic interventions. Hyperscanning experiments may be an essential part of it, as the ASD deficits are substantially deficits in social interaction, and it is critical to capture how individual-level temporal patterns translate into interpersonal coordination difficulties across development.

Notably, the high-sensitivity ASD profile described in this framework appears most consistent with the low-support-needs subgroup characterized by intact within-group communication (Crompton et al., 2025; Wilks et al., 2025). Other ASD subgroups may be characterized by other patterns (e.g., hypo-sensitivity), which would engage distinct mechanisms, potentially including reduced excitatory/inhibitory ratios leading to different synchrony patterns. Based on simulation analysis (Nguyen and Rubchinsky, 2021, 2024), changes in E/I balance can affect both the overall synchrony level and its temporal patterning, suggesting that different E/I profiles could underlie the heterogeneous sensory phenotypes observed across the autism spectrum. More research into ASD subgroups is needed to investigate dynamic patterns of neural activity characteristic of specific behavioral phenotypes.

The integration of recent neurodynamics research with social communication findings points toward a fundamental reconceptualization of autism. Rather than viewing autistic neural patterns as necessarily deficient, this research suggests they represent alternative organizational strategies with both adaptive advantages and environmental mismatches (Cockerham and Malaia, 2016; Tomeny et al., 2023). Understanding these patterns of neural activity with their subtle differences in the coordination of temporal dynamics through the lens of neurotype compatibility offers a path toward more effective interventions and a more nuanced appreciation of neurodiversity.
